# A New Species of *Demodex* (Acariformes: Prostigmata) Observed in the Mouflon, *Ovis aries musimon* (Artiodactyla: Bovidae) with Data on the Parasitism and Occurrence of Other Ectoparasites †

**DOI:** 10.3390/ani13162619

**Published:** 2023-08-14

**Authors:** Paulina Kozina, Joanna N. Izdebska, Leszek Rolbiecki

**Affiliations:** Department of Invertebrate Zoology and Parasitology, Faculty of Biology, University of Gdańsk, Wita Stwosza 59, 80-308 Gdańsk, Poland; paulina.kozina@ug.edu.pl (P.K.); leszek.rolbiecki@ug.edu.pl (L.R.)

**Keywords:** mouflon, *Ovis aries musimon*, *Demodex musimonis*, *Ixodes ricinus*, *Lipoptena cervi*, ectoparasites, skin mites

## Abstract

**Simple Summary:**

The aim of the study was to analyse the community of parasitic mites in mouflons *Ovis aries musimon* from the Polish population introduced from the Mediterranean area. It was important to determine whether these parasites were specific to or typical of the genus *Ovis* and whether foreign species had appeared with the mouflons that might pose a parasitological threat to the native fauna or, according to the theory of *parasites lost*, the introduction contributed to the loss of the original fauna of parasitic mites and the subsequent repopulation of the vacant microhabitats by species typical of local bovids, cervids, and other ungulates. Forty-one mouflons, obtained by hunting, were examined. Two species of mites were found, including *Demodex musimonis* sp. nov., previously unknown demodecid mites, probably specific to the mouflon. These discoveries were accompanied by the tick *Ixodes ricinus*, a parasite common in many parts of Europe with a wide host range, and *Lipoptena cervi*, a parasitic fly typical of ungulates. The new species represents the first finding of a skin mite of the family Demodecidae in wild representatives of the caprine Caprinae, and the present study is the first to examine the parasitic arthropods of the mouflon, including ectoparasites and skin mites. *Demodex musimonis* sp. nov. occurred asymptomatically in the mouflon, and as a specific species does not pose a threat to the native fauna. In contrast, the presence of *I. ricinus* clearly indicates a broadening of the reservoir for pathogens transmitted by epidemiologically significant ectoparasite species in Europe.

**Abstract:**

A greater understanding of mite biodiversity and ecology can explain their preference for microhabitats within host bodies, i.e., as ecto-, meso-, and endoparasites. Similarly, learning about the patterns formed by mite communities in wild animals can shed light on the mechanisms of parasitosis development in their domesticated counterparts. Hence, the present study examined the acarofauna of the mouflon, introduced to Poland in the early 20th century from a region of endemic occurrence. Forty-one individuals were examined for the presence of ectoparasites between 2010 and 2013; later, skin fragments were analysed for the presence of skin mites. A new species of Demodecidae, *Demodex musimonis* sp. nov., was discovered in the skin of the pasterns of 14.6% mouflons, as well as the ectoparasitic tick *Ixodes ricinus* (prevalence 29.3%) and ungulate-typical fly *Lipoptena cervi* (34.1%). However, no mites typical for ovine *Ovis* (Psorergatidae, Psoroptidae) were noted, nor any colonisation of microhabitats by species from local ungulates, resulting from the loss of original parasites during the introduction. A comparison of the acarofauna of the mouflon and domestic sheep *Ovis aries aries* suggests that a mite community with a complex structure and the co-occurrence of different families may be formed. However, it is not known whether the acarofauna of domestic sheep is derived from wild ancestors or the process of domestication. Even so, the peculiar Demodecidae must have been part of the natural parasitofauna of wild sheep, of which only *D. musimonis* sp. nov. has been recognised so far.

## 1. Introduction

An interesting direction for the study of mite biodiversity and ecology is the analysis of their occurrence in specific environments, which for parasites is the host’s body. Parasitic mites are able to make optimal use of the host by colonising all available microhabitats; most live as ectoparasites, residing in the fur and on the skin, but others colonise the skin as mesoparasites and others the respiratory tract or digestive system as endoparasites. Some stay on the host stationarily, throughout their lives, using it not only as a source of food and a place of shelter but also for the development of juvenile stages. Others use host resources periodically. Within a host, different mites can co-occur with each other, regardless of whether they are from the same group, with similar habitat requirements, or from different systematic and ecological groups differing in lifestyle, with varied food preferences, developmental cycles, adaptations, and variant parasitism. However, the co-occurrence of different mite species from the same families (e.g., Chirodiscidae, Demodecidae, and Psorergatidae) and even genera has mainly been described in rodents [1,2,3,4]. Similarly, most analyses of the co-occurrence of representatives from different systematic groups and parasitism types have concerned small mammals, e.g., [5,6,7,8,9,10]. Comparatively little data exists on co-occurrence and habitat sharing in the context of the ungulate mammalian Artiodactyla acarofauna. 

Among wild animals, more information has been obtained regarding the European bison *Bison bonasus* (Linnaeus, 1758) (Bovidae: Bovinae), in which 11 species of mites from the Ixodidae (Parasitiformes: Ixodida), Psoroptidae, Sarcoptidae (Acariformes: Astigmata), and Demodecidae (Acariformes: Prostigmata) have been found. However, no such studies are available for the wild representatives of the subfamily Caprinae, including the widely distributed genus *Ovis* in Eurasia and North America. So far, such data have only covered the domestic sheep *Ovis aries aries* Linnaeus, 1758, where both temporary parasitic mites (Ixodidae) and stationary skin mites of the Astigmata (Psoroptidae and Sarcoptidae) and Prostigmata (Demodecidae and Psorergatidae) have been recorded, including specific parasites of this host: *Demodex aries* Desch, 1986, *D. ovis* Railliet, 1895, and *Psorobia ovis* (Womersley, 1941). Most information, however, relates to observations of the disease manifestations of the parasitoses they cause (psoroptosis, chorioptosis, sarcoptosis, and demodecosis), whose development is often related to domesticated lifestyle and husbandry conditions [11,12,13,14,15,16]. In contrast, in the natural environment, such parasitoses are rare, and parasitic mites usually form stable and balanced host–parasite systems with their hosts, where load parasite levels do not exceed host tolerance levels and infestations do not cause disease symptoms [17]. In addition, breeding conditions favour infestations with atypical parasites that are passed from other mammals (neighbouring hosts) under favourable circumstances [18]. Therefore, there is undoubtedly more value in observing the biodiversity and co-occurrence of mites in mammals from wild populations. In addition, learning about the pattern of occurrence of the mite community in wild animals can further our understanding of the mechanisms of parasitoses development in their domesticated counterparts. 

In this sense, the mouflon *Ovis aries musimon* (Pallas, 1811) is a particularly interesting species and one whose acarofauna remains relatively unstudied. Its relict populations have inhabited certain Mediterranean islands, including Corsica and Sardinia, for several years. Mouflons have occasionally been introduced into various areas of Europe throughout both historical and modern times. At the beginning of the 20th century, they also found their way to the southern regions of Poland, where they are an alien species. Currently, their numbers in Poland are estimated at 3000 individuals, with the largest population, about 70% of all individuals, living in the Lower Silesia region [19,20]. 

Existing data on the parasitic mites of mouflon in its various regions of distribution has been limited to a few mentions of the occurrence of ticks [21,22]. Therefore, the purpose of the current study was to determine whether the mite community in mouflons from the Polish population includes specific species or at least those typical of the genus *Ovis*, especially members of the Demodecidae, Psorergatidae, and Psoroptidae. In addition, it aims to confirm whether the introduction of mouflons has brought with it alien species to the fauna of Poland that may pose a potential parasitological threat to the native fauna; it also examines whether, according to the theory of *parasites lost* [23], the introduction contributed to the loss of the natural/primary acarofauna in the mouflon, leaving vacant microhabitats that could be populated by species typical of local ungulates, such as those of the roe deer *Capreolus capreolus* (Linnaeus, 1758). The current study included all ectoparasites, including insects, to account for the possibility of correlation or interaction with parasitic mites sharing the same or neighbouring microhabitats.

## 2. Materials and Methods

A total of 41 European mouflons, *Ovis aries musimon*, from Poland, Lower Silesia, were examined, including 19 from the Jemna Forestry (Bardo Forest Inspectorate; 50°35′35.97″ N, 16°39′4.28″ E) and 22 from the Jugów Forest Inspectorate (near Sokolec; 50°39′11.69″ N, 16°28′24.25″ E). The animals were obtained between October 2010 and November 2013 from shoots carried out by employees of the forestry districts or from foreign exchange shoots. Ectoparasites living on the body surface and in the fur were collected immediately after shooting [24]. In addition, skin fragments were taken from various areas of the body, including the head/ear pinnae, neck, back, abdomen, groin, and pasterns. These fragments were preserved in a 70% ethanol solution for later studies of skin mite occurrence. For the recovery of demodecid mites, skin fragments were individually digested in a 10% potassium hydroxide solution as described previously [25]. The digested material was decanted and examined under phase-contrast microscopy (Nikon Eclipse 50i) with one 1 cm^2^ of skin sample yielding approximately 100 wet preparations. The mites were placed in a polyvinyl-lactophenol solution and the following measurements were taken: total body length = length of gnathosoma, podosoma, and opisthosoma; gnathosomal width (at base); and podosomal and opisthosomal width (maximum width). All measurements are given in micrometres.

The specimen depositories are cited using the following abbreviation: UGDIZP, University of Gdańsk, Department of Invertebrate Zoology and Parasitology, Gdańsk, Poland [26]. 

The description of the species adopted the nomenclature commonly used for the family Demodecidae [27] and was completed with the nomenclature proposed by Bochkov [28] for the superfamily Cheyletoidea (Acariformes: Prostigmata) and by Izdebska and Rolbiecki [4]. The scientific and common names of the hosts follow Wilson and Reeder [29] and the Integrated Taxonomic Information System [30]. Basic parasitological parameters, i.e., the prevalence (percentage of hosts infected), mean intensity (mean number of parasites in infected hosts), intensity range (minimum and maximum number of parasite individuals per host), mean density (mean number of parasites per unit area; counted only for demodecid mite), and density range (minimum and maximum number of parasite per unit area; counted only for demodecid mite) were measured to determine the host infection level [31].

## 3. Results

### 3.1. Overall Mouflon Infestation

**Figure 1:**
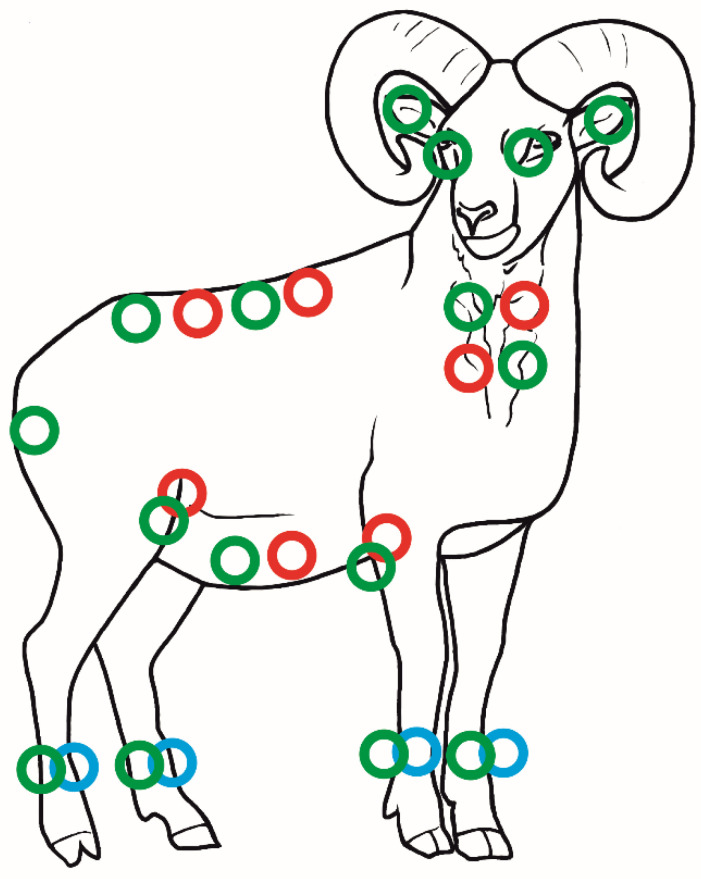
Topography preferences of ectoparasites on the mouflon: ● *Ixodes ricinus*, ● *Lipoptena cervi*, and ● *Demodex musimonis* sp. nov.

Two species of parasitic mites were found—*Ixodes ricinus* Linnaeus, 1758 (41 ♀♀, 31 ♂♂, 6 NN, and 1 L) and *Demodex musimonis* sp. nov. (23 ♀♀, 16 ♂♂, and 2 DN). Among the other parasitic arthropods, the fly *Lipoptena cervi* (Linnaeus, 1758) was also found (37 ♀♀, 25 ♂♂, and 1 egg). The parasites showed a strict topographical preference, with the majority of ticks (53.2%) recorded on the neck, and the remainder on the abdomen (13.9%), groin (11.4%), back (8.9%), anal area (2.5%), eye (2.5%), pastern (2.5%), and ear (1.3%); for three individuals (3.8%), the location was not determined. Also, *L. cervi* preferred the neck area (48.3%), while the others preferred the back (22.6%), abdomen (19.4%), and groin (9.7%). In turn, all specimens of *D. musimonis* sp. nov. occurred in the skin of the pastern (Figure 1).

The overall prevalence (including all parasite species) was 56.1%. In turn, the infestation of *I. ricinus* was 29.3%, 6.6, 1–43, for *L. cervi* 34.1%, 4.4, 1–23, and for *D. musimonis* sp. nov. 14.6%, density of 6.8/1 cm^2^, density range of 3–16/1 cm^2^.

### 3.2. Descriptions (Figure 2 and Figure 3, Table 1)

*Demodex musimonis* Izdebska, Kozina and Rolbiecki, 2023.

Male (*n* = 15 and holotype). Body elongated, stocky, conical, distinctly separated gnathosoma; 196 (183–207) long and 43 (38–47) wide (holotype, 196 × 43). Gnathosoma trapezoidal, slightly shorter than width at base; on dorsal side in the central part of basal segment, pair of wedge-shaped (rounded at end, posterior edge with bulge) supracoxal spines (setae *elc.p*) present, ca. 4.0 long (holotype, 4.0), directed medially, oblique. Palps three-segmented, terminating in three different shaped spines (one smallest—single, unbifurcated, conical; two bifurcated—larger and smaller) on tibio-tarsus; also, small setae *v”F* present on middle segment (trochanter-femur-tarsus). On ventral surface of gnathosoma, horseshoe-shaped pharyngeal bulb with pair of conical subgnathosomal setae (setae *n*), situated anterior on both sides. Podosoma rectangular; four pairs of short legs with coxa integrated into ventral idiosomal wall and five free, overlapping segments (trochanter-tarsus); two bifurcated claws, ca. 5.0 long (holotype, 5.0), with pointed subterminal spur on each tarsus; knob on each femur. Epimeral plates (coxal fields) distinctly sclerotized; all trapezoidal. On the dorsal side of podosoma, podosomal shield with distinctly irregular striation, reaches level of legs III; posterior edge of this shield is convex. Opisthosoma constitutes 63% (59–66%) of body length (holotype, 62%); conical, wide at base, slightly rounded at end. Whole opisthosoma distinctly annulated; annulations reach dorsal podosoma side (pair of legs III); annuli relatively wide at ca. 1.0–1.5 µm. Opisthosomal organ not visible. Aedeagus 22 (18–26) long, on dorsal surface, located at level of epimeral plates I–III. Genital opening located at level of posterior edge of epimeral plate I (on border with epimeral plate II).

**Figure 2:**
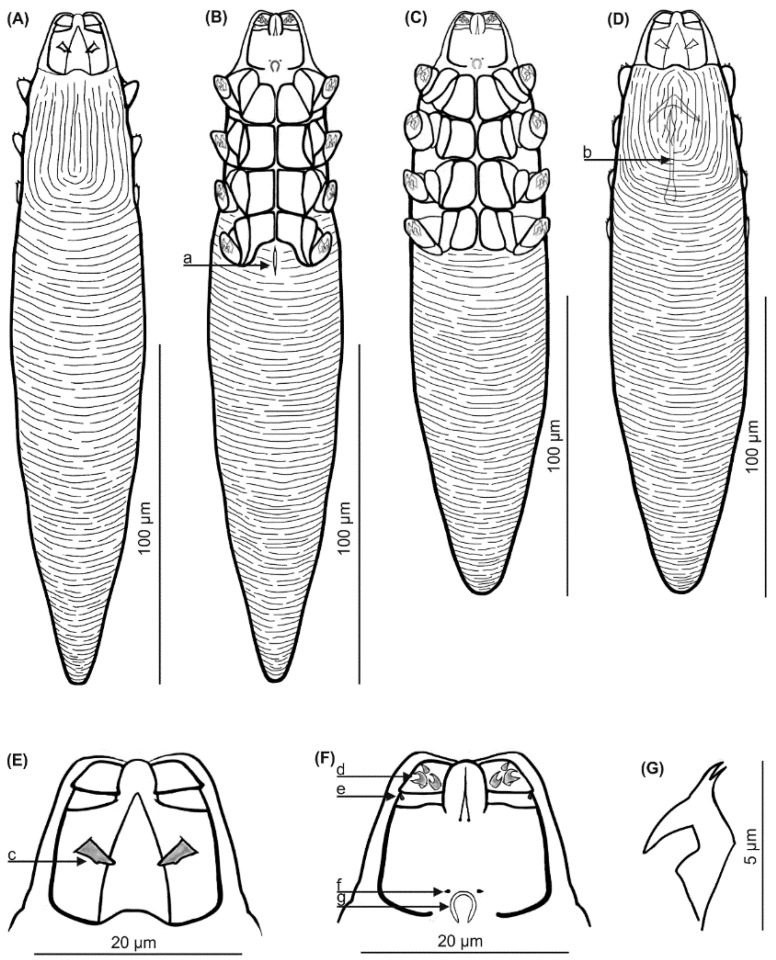
*Demodex musimonis* sp. nov.: female, dorsal view (**A**), female, ventral view (**B**), male, ventral view (**C**), male, dorsal view (**D**), gnathosoma, male, dorsal view (**E**), gnathosoma, male, ventral view (**F**), claw on the leg (**G**); a: vulva, b: aedeagus, c: supracoxal spine (seta *elc.p*), d: spines on palps, e: seta *v”F*, f: subgnathosomal seta (seta *n*), and g: pharyngeal bulb.

**Figure 3:**
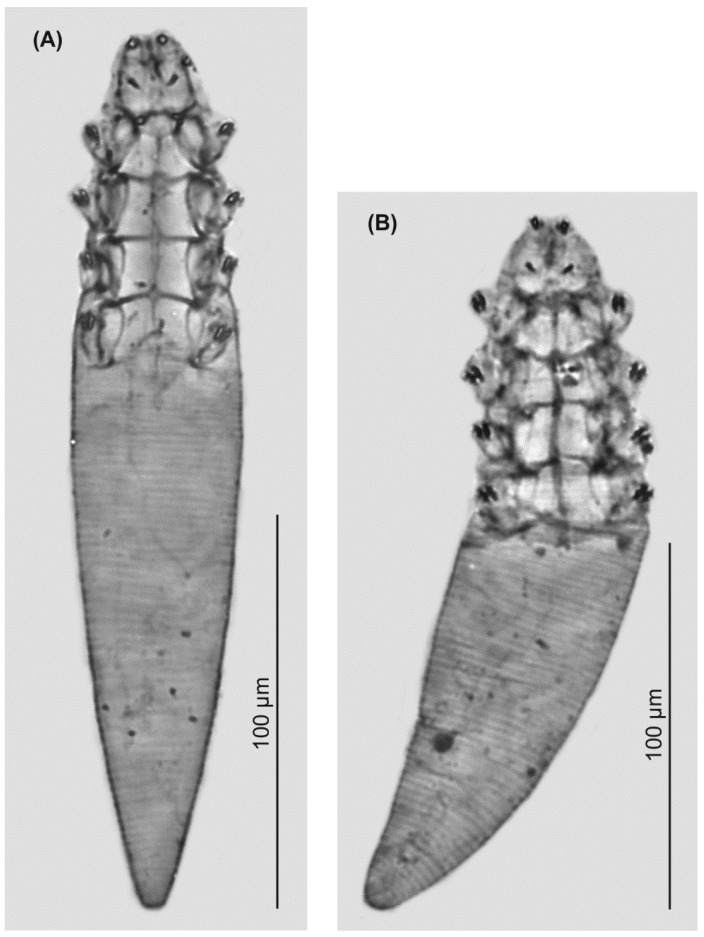
*Demodex musimonis* sp. nov.: female (**A**), male (**B**).

**Table 1:** Body size (micrometres) for adults of *Demodex musimonis* sp. nov.

Morphological Features	Males (*n* = 16) Mean (Range) ± SD	Females (*n* = 23) Mean (Range) ± SD
Length of gnathosoma	18 (17–20), ±1	19 (18–20), ±1
Width of gnathosoma (at base)	21 (20–23), ±1	22 (20–24), ±1
Length of podosoma	55 (51–59), ±2	66 (60–73), ±4
Width of podosoma	43 (38–47), ±2	44 (38–47), ±2
Length of opisthosoma	123 (108–131), ±6	150 (130–172), ±13
Width of opisthosoma	41 (38–45), ±2	44 (42–47), ±1
Aedeagus	22 (18–26), ±2	–
Vulva	–	12 (10–14), ±1
Total length of body	196 (183–207), ±6	236 (208–257), ±14

SD, standard deviation.

Female (*n* = 23). Body elongated, more slender than male, spindle-shaped, distinctly separated gnathosoma; 236 (208–257) long and 44 (42–47) wide. Gnathosoma shape similar to male. Pharyngeal bulb and morphological details of gnathosoma similar to those in male. Podosoma conical, slightly widening posterior end; legs similar to those in male. Epimeral plates distinctly sclerotized; I pair trapezoidal, II–III rectangular; posterior edges of pair IV form distinctly incision. On the dorsal side of podosoma, podosomal shield, with distinctly irregular striation, reaches level of legs III; posterior edge of this shield is convex. Opisthosoma constitutes 64% (61–69%) of body length; conical, rounded at end. Whole opisthosoma distinctly annulated; annulations reach dorsal podosoma side (pair of legs III); annuli relatively wide at ca. 1.0–1.5 µm. Opisthosomal organ not visible. Vulva 12 (10–14) long, located slightly behind incision of IV epimeral plates.

Egg (*n* = 1): 70 long and 22 wide.

Deutonymph (*n* = 2): 85–235 long and 20–40 wide.

Material deposition: Male holotype (reg. no. UGDIZPBOamDDm04m), 15 male paratypes (reg. no. UGDIZPBOamDDm01m–03m, UGDIZPBOamDDm05m–16m) and 23 female paratypes (reg. no. UGDIZPBOamDDm01f–23); pastern region; host *Ovis aries musimon* (reg. no. MABOam10/2012, MABOam18/2011, MABOam33/2012, MABOam34/2013, MABOam37/2013, and MABOam41/2013); Sokolec and Jemna, Poland; January 2011, November 2012, January 2012 and November 2013; coll. J.N. Izdebska and P. Kozina; the whole-type material (mounted microscope slides with the demodecid mites) deposited within the framework of the Collection of Extant Invertebrates in Department of Invertebrate Zoology and Parasitology, University of Gdańsk, Poland.

Location in the host: *Demodex musimonis* sp. nov. was found exclusively in the pastern region of the examined mouflon.

Etymology: The specific epithet *musimonis* refers to the subspecific name of the host.

Differential diagnosis: the shape of *Demodex musimonis* sp. nov., and some of its features, most closely resemble those of *D. ovis* Hirst, 1919 (redesc. Desch 1986) from the domestic sheep *Ovis aries aries* (Table 2). However, *D. musimonis* sp. nov. is a little larger and has different body proportions. In addition, *D. musimonis* sp. nov. demonstrates a more distinct sexual dimorphism: the body length-to-width ratios being 4.6 in males and 5.3 in females; in contrast, in *D. ovis*, both sexes demonstrate similar proportions (5.2 for males and 5.5 for females). Supracoxal spines on the gnathosoma are large (4 µm) and wedge-shaped in *D. musimonis* sp. nov., while they are smaller (2 µm) and peg-like in *D. ovis*. The spines on the terminal segment of the palpi are similarly shaped in both species but are different in size and more massive in *D. musimonis* sp. nov.; moreover, *v”F* seta are present on the palpi of *D. musimonis* sp. nov. Subgnathosomal setae in *D. musimonis* sp. nov. are located relatively higher (at the level of anterior margin of the pharyngeal bulb) than in *D. ovis* (below the anterior margin of the pharyngeal bulb). The epimeral plates are distinctly sclerotized in *D. musimonis* sp. nov., with clearly outlined edges, but are weakly sclerotized (edges indistinct) in *D. ovis*. In addition, the posterior edges of the epimeral plate IV in *D. musimonis* sp. nov. females possess a large, trapezoidal incision encompassing a vulva, while they are weakly outlined in female *D. ovis*, with a small triangular incision, below which the vulva is located. Furthermore, *D. musimonis* sp. nov. males demonstrate a longer aedeagus (mean 22.0 µm in length) located at level of epimeral plates I–III, with the genital opening at the level of the posterior part of epimeral plate I; in male *D. ovis*, the aedeagus is shorter (mean, 20.0 µm in length) and located at epimeral plates II–III with the genital opening at the level of epimeral plate II. The typical microhabitat is also different: *D. musimonis* sp. nov. was exclusively found in the pastern region, while *D. ovis* was noted throughout the entire body, though preferring the head, flanks, and shoulders. 

## 4. Discussion

Undoubtedly, the most important result of the current research is the discovery and description of *Demodex musimonis* sp. nov., a new species of Demodecidae associated with the mouflon, and one which may be a specific parasite. The Demodecidae exhibit high host specificity, with several monoxenic species often found in individual mammals; they are known to inhabit different microhabitats within the skin or other host structures and tissues [17]. The largest number of such synhospital species has been recognised in rodents, such as seven in the house mouse *Mus musculus* Linnaeus, 1758. However, such coexistence has also been noted among ungulates: three species have been described in domestic cattle *Bos taurus* Linnaeus, 1758, and two each in domestic sheep, European bison, red deer *Cervus elaphus* Linnaeus, 1758, and the domestic horse *Equus caballus* Linnaeus, 1758 [17]. Host-specific demodecid mites, especially those found in wild mammals, often show a high intensity or density of infection while lacking symptoms of parasitosis; as such, they usually accompany their hosts to different regions of occurrence and can be introduced with them to new regions [17,33,34]. However, due to their high peculiarity, they do not present significant threats to native fauna as alien species.

The demodecid mites noted in the present study showed topographical specificity, colonising only the pastern region. Such preference is a phenomenon typical of mites of this group, where only some species (associated with follicles of normal hair) colonise the entire skin surface, albeit usually unevenly [35]. Examples include *D. kutzeri* Bukva, 1987, found in red deer, which has been recorded in skin fragments from more than half of the studied deer, or *D. ovis* from domestic sheep [36,37,38,39]. Most species are found in narrow microhabitats, often concentrated in the head region, such as the Meibom’s glands of the eyelids (e.g., *D. bisonianus* Kadulski and Izdebska, 1996 from European bison, *D. ghanensis* Oppong, Lee, and Yasin, 1975 from domestic cattle), the eye region (e.g., *D. tauri* Bukva, 1986 from domestic cattle), the hairless skin region of the nose (*D. bialoviensis* Izdebska, Rolbiecki and Bielecki, 2022 from European bison), the ear canals (e.g., *D. conicus* Izdebska and Rolbiecki, 2015 from a house mouse), the vibrissae region (e.g., *Miridex putorii* Izdebska, Rolbiecki, and Rehbein from the polecat *Mustela putorius* Linnaeus, 1758), and the tongue and gums (*Glossicodex musculi* Izdebska and Rolbiecki, 2016 from a house mouse) [4,39,40,41,42,43,44]. A few species of Demodecidae are known to be restricted to the limb region. Examples include *D. obliquus* Izdebska and Rolbiecki, 2022 from the domestic cat *Felis catus* Linnaeus, 1758, or *D. ponderosus* Izdebska, and Rolbiecki, 2014 from the Norway rat *Rattus norvegicus* (Berkenhout, 1769) [45,46]. 

In the present study, *D. musimonis* sp. nov. demonstrated a relatively low level of infestation (prevalence 14.6%), with only 41 specimens being found in the studied skin fragments. This may be due to the locality of occurrence (pastern), the survey technique, i.e., the labour-intensive method of digesting mites from selected skin fragments, and also the dispersal (low density of mites in the skin). It is also possible that the study period was not optimal for population development, as indicated by the discovery of only a few juvenile stages. 

Among those infecting the genus *Ovis*, demodecid mites have only been described in domestic sheep. *Demodex ovis*, discovered first in sheep, can occur throughout the whole body, preferring the region of the head and trunk, while *D. aries*, described in the late 20th century, is associated with areas of high sebaceous gland density (e.g., the skin around the vulva, foreskin, and nostrils) [32]. In addition, Bukva [36] mentions yet another unidentified species of *Demodex* found in the head/eyelid region. 

It is difficult to compare the Demodecidae from the domestic sheep with the demodecid mite described from the mouflon. Although *D. musimonis* sp. nov. morphologically resembles *D. ovis*, it shows different topographical preferences. Perhaps the species share a common pedigree. For such an inference, however, the pedigree of the mouflon itself is problematic, being a wild sheep that is believed to have inhabited the Mediterranean island region for 6000 to 8000 years (according to various sources), and probably originally originated from Asia. However, the origin of mouflons in Europe is controversial, and the pedigree of domestic sheep has not yet been conclusively clarified; it is possible that the mouflon may be a protoplast or one of several possible ancestors [47,48,49,50,51]. In general, questions of the evolution of host–parasite systems in the context of *Ovis*–*Demodex* require more extensive analyses of material from different hosts and regions of occurrence.

Despite this valuable discovery, the acarofauna of mouflons from Poland should be considered poor; in a representative sample of 41 hosts examined in different seasons, only two species of mites were found. In addition, to the representative of the Demodecidae, a new discovery, only the polyxenic tick *Ixodes ricinus*, common in various regions of Europe, was recorded. Other parasitic arthropods were also found to be poorly represented: only *Lipoptena cervi*, an oligoxenic ectoparasite of ungulates, especially cervids, usually found in large numbers in autumn, was observed [21,52]. The hematophagous temporal ectoparasites, castor bean ticks, and deer flies showed similar topographical preferences, i.e., they often occurred in the neck region, which is probably related to the availability of blood vessels [33]. 

The significance of the parasites found among the mouflons is difficult to determine. *Demodex musimonis* sp. nov. occurred sparsely and asymptomatically, which is typical of such mite infestations among wild mammals, e.g., [53,54]. *Ixodes ricinus*, on the other hand, probably had no pathogenic significance as a parasite, but its role in mouflon with regard to pathogen transmission is unknown. Undoubtedly, the mouflon represents an increase in reservoir animals for pathogens transmitted by *I. ricinus*, regarded as an ectoparasite with considerable epidemiological significance in Europe.

However, no representatives of the Psorergatidae, Psoroptidae, Sarcoptidae, or even other Ixodidae, which frequently attack ungulates, were found in the mouflon, nor any typical ungulate parasitic insects from the Phthiraptera. This is not a matter of the limited sample size or study period. Analogous observations, using the same research methods, carried out on other ungulates reported a much richer community of mites inhabiting body surfaces and skin. A good object for such comparisons seems to be the European bison, which, like the mouflons found in Poland, was introduced into the environment through human activities. The European bison became extinct under natural conditions at the beginning of the 20th century, and the current restitution of the species resulted from reintroductions or introductions of individuals that survived in breeding conditions and zoos. To date, 14 species of parasitic arthropods have been found in the European bison (considering only parasites *sensu stricto*), including 11 species of mites, including 2 specific Demodecidae, as well as oligoxenic, ungulate-typical Psoroptidae, a polyxenic species of Sarcoptidae and various Ixodidae. In European bison, mites or other parasitic arthropods are recorded in all seasons (most of the studies were conducted in winter), and the prevalence is sometimes high; hence, studies of even a small sample yield findings of at least several species. For example, surveys of 12 bison from the Bieszczady Mountains conducted in the winter from 1998 to 2000 yielded findings of four species of parasitic arthropods (including three mites), while winter surveys of 12 European bison from a reserve in the Bialowieza Forest yielded nine species (including seven mites). Surveys of just six bison conducted in July 2011 yielded the identification of seven species of parasitic arthropods (including six species of mites) [32,33,34]. However, European bison, apart from the natural specific (monoxenic) parasitofauna preserved in this mammal (including two species of the Demodecidae), could take over the parasitofauna from other ungulates, probably cervids and cattle, during the restoration of the wild populations [33]. Hence, they harbour species of Psoroptidae typical of the Bovidae—*Chorioptes bovis* (Hering, 1845), *Psoroptes equi* (Hering, 1838), the polyxenic *Sarcoptes scabiei* (DeGeer, 1778), or ticks associated with local environments, particularly *I. ricinus* and *Dermacentor reticulatus* Fabricius, 1794. However, *Ch. bovis* or *P. equi* (syn. *P. ovis*—according to Zahler et al. [55]) are also typical parasitofauna of domestic sheep [56], so they could potentially be parasites of natural mouflon populations. 

In addition, if any parasites are lost in the introduction process, they could be taken over by local ungulates, such as the roe deer or red deer found in the region, in which these mites are common [52]. Here, however, it should be remembered that the takeover of skin mites has many limitations. Not only does it depend on the ability of the parasite to colonise and adapt to another host, but also on the direct contact between hosts needed for transfer. Interspecies contact is relatively rare and involves, for example, mammalian carnivorans (predator–prey contact). 

The presence of other skin mites in the mouflon would undoubtedly shed light on the findings of such mites in mouflons from other populations. Unfortunately, there is a lack of data on parasitic arthropods of mouflons from other areas of distribution, including endemic areas, apart from a few reports of the presence of local tick species [21,22,57,58].

## 5. Conclusions

A comparison of the acarofauna of the mouflon with that of the domestic sheep suggests the potential formation of a mite community with a complex topical and topographical structure, with the possibility of co-occurrence of representatives of several skin mite families. It is not known, however, whether this structure was adopted by domestic sheep from wild ancestors or formed through domestication, where oligoxenic Psoroptidae may have been adopted from other domesticated ungulates. However, the typically monoxenic/specific Demodecidae, or Psorergatidae, should already constitute the natural parasitofauna of wild sheep. 

The current study has so far yielded the discovery of one species from these mite groups associated with wild sheep (mouflons): *D. musimonis* sp. nov. In addition, this is the first finding of mites from the Demodecidae in wild Caprinae; so far, only two species have been described from the domestic sheep and one from the domestic goat, *Capra hircus* Linnaeus, 1758. It is also the first comprehensive study of parasitic arthropods inhabiting the mouflon, including both typical ectoparasites and skin mites.

## Figures and Tables

**Table 2:** Morphometric comparison between *Demodex musimonis* sp. nov. and *Demodex ovis*.

Feature/Species	*Demodex musimonis* sp. nov.		*Demodex ovis*	
Source	Present Study		Desch [32]	
Sex	Males	Females	Males	Females
Sample Size	(*n* = 16)	(*n* = 23)	(*n* = 20)	(*n* = 20)
Body total length	196 (183–207), SD 6	236 (208–257), SD 14	170 (140–201), SD 17 *	214 (187–274), SD 26 *
Body total width	43 (38–47), SD 2	44 (42–47), SD 1	33 (30–38), SD 3 *	39 (36–52), SD 4 *
Body length-to-width ratio	4.6:1 (3.9–5.1:1), SD 0.3:1	5.3:1 (4.5–5.8:1), SD 0.3:1	5.2:1 **	5.5:1 **
Opisthosoma length to body length ratio (%)	63 (59–66), SD 2	64 (61–69), SD 2	59 **	59 **
Aedeagus length	22 (18–26), SD 2	–	20 (18–22), SD 1 *	–
Vulva length	–	12 (10–14), SD 1	–	6 (4–7), SD 1

SD: standard deviation. * Measurements were rounded to the nearest micrometre with respect to the original results [32]. ** Calculated from measurements by Desch [32].

## Data Availability

The data presented in this study are available on request from the corresponding author.
